# Supplementary material for: The Evolution and Characterization of the RNA Interference Pathways in Lophotrochozoa
Source: Genome Biol Evol. 2024 May 7;16(5):evae098. doi: 10.1093/gbe/evae098 (PMC11114477; doi:10.1093/gbe/evae098)
Supplement: evae098_Supplementary_Data [file evae098_supplementary_data.zip › Supplementary_materials.zip!/selected_miRNAs/NoGe_selected_miRNAs/mirdeep_pdf_files/NMRB01000449.1_Notospermus_geniculatus_strain_Ushimado_scaffold449,_whole_genome_shotgun_sequence_13615.pdf]

Provisional ID	:	NMRB01000449.1_Notospermus_geniculatus_strain_Ushimado_scaffold449,_whole_genome_shotgun_sequence_13615
Score total	:	258
Score for star read(s)	:	3.9
Score for read counts	:	249.9
Score for mfe	:	1.1
Score for randfold	:	0
Score for cons. seed	:	3
Total read count	:	502
Mature read count	:	487
Loop read count	:	0
Star read count	:	15

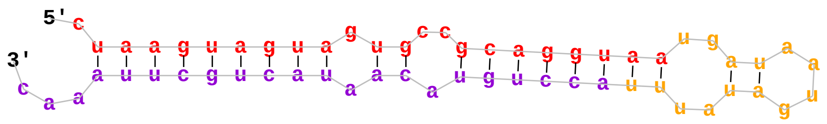

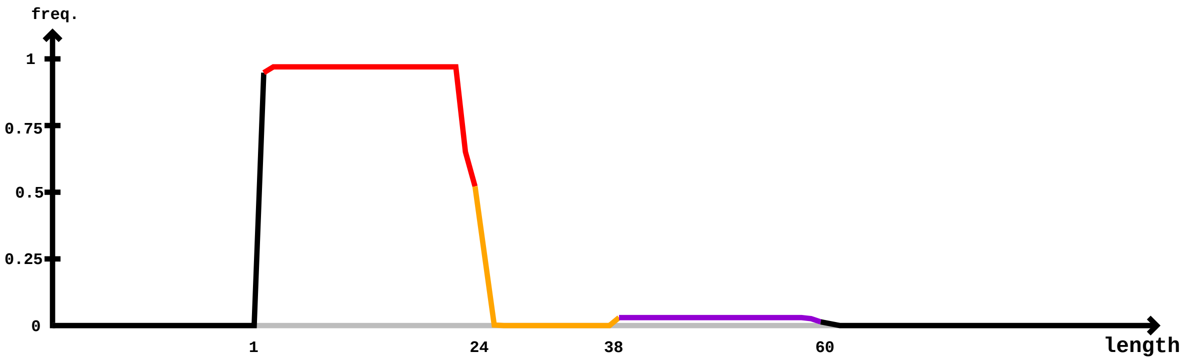

Mature Star

```
5'- aguuggcguuauugucucgacuaaguaguagugccgcagguaaugauaaugauauuuaccuguacaauacugcuuaaacgagcugaugaccauaucgaccugcucacgucaag  -3'   obs
    aguuggcguuauugucucgacuaaguaguagugccgcagguaaugauaaugauauuuaccuguacaauacugcuuaaacgagcugaugaccauaucgaccugcucacgucaag         exp
    ...((((((((..(.((((..(((((((((.((..((((((((..((....))..)))))))).)).)))))))))..))))))))))).)))....(((........)))...    reads   mm   sample
    ....................cuaaguaguagugccgcaggG........................................................................     1      1    Fm2
    ....................cuaaguaguagugccgcaggu........................................................................     22     0    Fm2
    ....................cuaaguaguagugccgcagguaa......................................................................     15     0    Fm2
    ....................cuaaguaguagugccgcagguaU......................................................................     13     1    Fm2
    ....................cuaaguaguagugccgcagguaUu.....................................................................     19     1    Fm2
    .....................uaaguaguagugccgcagguU.......................................................................     10     1    Fm2

    ....................cuaaguaguagugccgcaggu........................................................................     31     0    Fm1
    ....................cuaaguaCuagugccgcaggu........................................................................     11     1    Fm1
    ....................cuaaguaguagugccgcCggu........................................................................     1      1    Fm1
    ....................cuaaguaguagugccgGaggu........................................................................     1      1    Fm1
    ....................cuaaguaguagugccgcaggua.......................................................................     36     0    Fm1
    ....................cuaaguaguaAugccgcaggua.......................................................................     1      1    Fm1
    ....................cuaaguaguaCugccgcaggua.......................................................................     1      1    Fm1
    ....................cuaaguaguagugccgcagAuaa......................................................................     1      1    Fm1
    ....................cuaaguaguagugccgcagguaU......................................................................     13     1    Fm1
    ....................cuaaguaguagugccgcagguaa......................................................................     62     0    Fm1
    ....................cuaaguaguagugcUgcagguaa......................................................................     6      1    Fm1
    ....................cuaaguaguagugccgcGgguaau.....................................................................     1      1    Fm1
    ....................cuaaguaguagugccgcagguaau.....................................................................     19     0    Fm1
    ....................cuaaguaguagugccgcagguaUu.....................................................................     15     1    Fm1

    ....................cuaaguaCuagugccgcaggu........................................................................     14     1    Mm1
    ....................cuaaguGguagugccgcaggua.......................................................................     1      1    Mm1
    ....................cuaaguaguagugccgcaggua.......................................................................     1      0    Mm1
    ....................cuaaguaguagugccgcagguaa......................................................................     7      0    Mm1
    .....................uaaguaguagugccgcagguaU......................................................................     1      1    Mm1

    ....................cuaaguaCuagugccgcaggu........................................................................     13     1    Mm2
    ....................cuGaguaguagugccgcaggu........................................................................     1      1    Mm2
    ....................cuaaguaguagugccgcaggu........................................................................     13     0    Mm2
    ....................cuaaguaguagugccgcaggCa.......................................................................     1      1    Mm2
    ....................cuaaguaguagugccgcagguU.......................................................................     2      1    Mm2
```

```
                    Mature                               Star
aguuggcguuauugucucgacuaaguaguagugccgcagguaaugauaaugauauuuaccuguacaauacugcuuaaacgagcugaugaccauaucgaccugcucacgucaag
....................cuaaguaguagugccgcaggua.......................................................................   5    0   Mm2
....................cuaaguaguagugccgcagguaU......................................................................   7    1   Mm2
....................cuaaguaguagugccgcagguaa......................................................................   19   0   Mm2
....................cuaaguaguagugccgcGgguaau.....................................................................   1    1   Mm2
....................cuaaguaguagugccgcagguaUu.....................................................................   1    1   Mm2
....................cuaaguaguagugccgcagguaau.....................................................................   6    0   Mm2
....................cuaaguaguagugccgAagguaau.....................................................................   1    1   Mm2
....................cuaaguaguagugccgcagguaauU....................................................................   1    1   Mm2

....................cuaaguaguagugccgcaggu........................................................................   16   0   Fm3
....................cuaaguaCuagugccgcaggu........................................................................   10   1   Fm3
....................cuaagGaguagugccgcaggu........................................................................   1    1   Fm3
....................cuaaguaguagugccgcaggua.......................................................................   3    0   Fm3
....................cuaaguaguagugccgcagguaa......................................................................   25   0   Fm3
....................cuaaguaCuagugccgcagguaa......................................................................   2    1   Fm3
....................cuaaguaguagugccgcagguaaA.....................................................................   2    1   Fm3
....................cuaaguaguagugccgcagguaUu.....................................................................   1    1   Fm3
....................cuaaguaguagugccgcagguaau.....................................................................   5    0   Fm3
.........................................................accuguacaauacugcuuaa....................................   2    0   Fm3
.........................................................accuguacaauacugcuuaaa...................................   6    0   Fm3
.........................................................accuguacaauacugcuuaaac..................................   7    0   Fm3

....................cuaaguaCuagugccgcaggu........................................................................   13   1   Mm3
....................cuaaguaguagugccgcaggu........................................................................   12   0   Mm3
....................cuaaguaguagugccgcagguU.......................................................................   1    1   Mm3
....................cuaaguaguagugccgcaggua.......................................................................   3    0   Mm3
....................cuaaguaguagugccgcagguaa......................................................................   10   0   Mm3
....................cuaaguaguagugccgcagguaU......................................................................   2    1   Mm3
....................cuaaguaguagugccgcagguaUu.....................................................................   2    1   Mm3
....................cuaaguaguagugccgcagguaau.....................................................................   3    0   Mm3
....................cuaaguaguagugccgcagguaaA.....................................................................   2    1   Mm3
```
